# Nasolabial Pinch Airway Seal to Enable Positive-Pressure Ventilation: 40 Years of Expanding Use

**DOI:** 10.31486/toj.21.0051

**Published:** 2021

**Authors:** James M. Riopelle, Nichole C. Staab, John N. Cefalu, Mark J. Wilson

**Affiliations:** ^1^ Department of Anesthesiology, LSU Health Sciences Center–New Orleans, New Orleans, LA; ^2^ Department of Anesthesia, University Medical Center–New Orleans, New Orleans, LA

## TO THE EDITOR

For more than 40 years, our department's anesthesia instructors have employed nasolabial pinch airway seal (NLPAWS) to give trainees additional time to perform nasotracheal intubation. When a trainee is unable to advance the tip of the tracheal tube through the vocal cords and the patient's pulse oximeter signals declining oxygen saturation (SpO_2_), the trainee is instructed to do the following:

1. Leave the endotracheal tube (ETT) tip immediately supraglottic.
2. Withdraw the laryngoscope.
3. Connect the breathing circuit to the ETT adapter.
4. Use the left hand to pinch closed the patient's lips and nose around the ETT.
5. With the right hand, initiate positive-pressure ventilation (PPV) using the anesthesia circuit bag.

This oxygenation technique has proved consistently successful provided (1) the ETT tip is immediately supraglottic (retro-epiglottic) and (2) glottic closure (laryngospasm) has been prevented by prior administration of a muscle relaxant drug.

In the late 1990s, members of our department began employing NLPAWS to ventilate adult patients (following anesthetic induction or cardiac arrest) via an orally inserted tracheal tube when PPV could not be administered because of a large leak at the facemask-skin interface. Such occurrences most often resulted from (1) the patient having a full beard or sunken cheeks, (2) the anesthetist having small hands or a lack of experience, or (3) use of a missized facemask (Figure).

**Figure.**
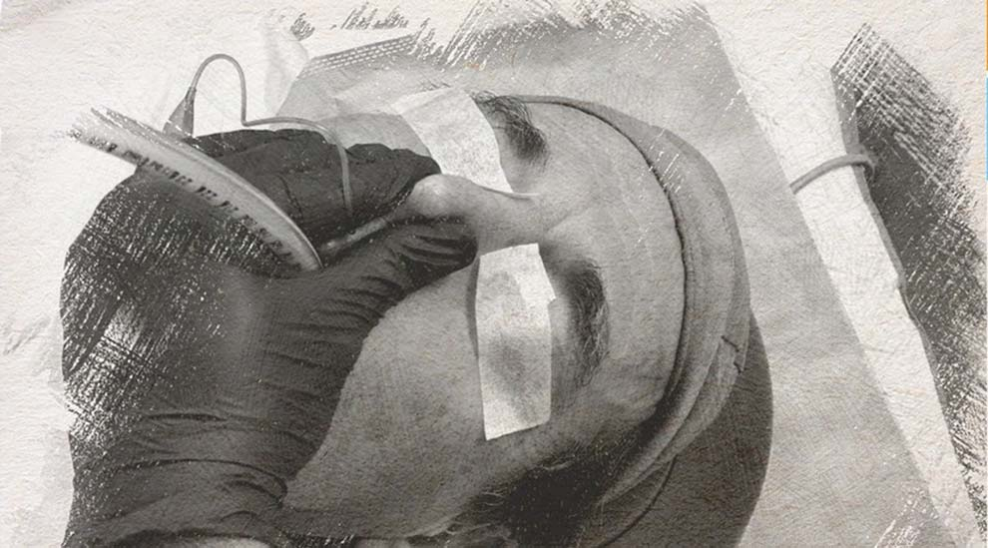
**Simulation of one-handed application of nasolabial pinch airway seal in a volunteer.** (Photo credit to Brian M. Klamer, CRNA, DNP, staff nurse anesthetist, Department of Anesthesia, University Medical Center–New Orleans, New Orleans, LA)

At first, we used NLPAWS to permit ventilation through an oral ETT inserted a distance approximating the length of an appropriately sized oral airway.^1^ In approximately 20% of these cases, however, PPV failed because of airway obstruction (*not* air leak). A solution to this problem was found to be using a laryngoscope to guide the ETT tip to the same immediately supraglottic position that had always been a part of NLPAWS use during nasotracheal intubation.^2^ Two advantages of this technique of rescue ventilation compared to similar use of a laryngeal mask airway (LMA) are (1) faster access to an ETT and (2) suitability of almost any ETT size.

Very recently, members of our department have learned to use NLPAWS to enable PPV (1) in the presence of a leaking LMA (5 cases); (2) via a nasal airway fitted with an ETT adaptor during upper GI endoscopy (7 cases, all by one anesthetist whose large hands probably helped him minimize air leak around the endoscopy bite block); and (3) via a correctly inserted ETT (4 cases). In 3 cases in the last category, the problem was simply that that the air syringe reserved for inflating the ETT cuff had been misplaced. In the fourth case, however, NLPAWS use may have been lifesaving. ETT cuff rupture occurred (probably from contact with a prominent incisor) during intubation of a patient with severe COVID-19 pneumonia. NLPAWS enabled sufficient Ambu ventilation (100% O_2_ and PEEP 20 cm H_2_O) to reverse a rapid decline in blood oxygenation (SpO_2_ 88% → 42%) and elevate it to 58% during the time required for a replacement ETT to be brought to the patient's negative-pressure intensive care unit room.

We are not aware of any instance of gastric aspiration occurring during NLPAWS use by a member of our department. Risk of this complication may be similar to risks associated with PPV performed using a facemask plus oral airway.
